# Decreased degree centrality values as a potential neuroimaging biomarker for migraine: A resting-state functional magnetic resonance imaging study and support vector machine analysis

**DOI:** 10.3389/fneur.2022.1105592

**Published:** 2023-01-30

**Authors:** Qian Wang, Yujun Gao, Yuandong Zhang, Xi Wang, Xuying Li, Hang Lin, Ling Xiong, Chunyan Huang

**Affiliations:** ^1^Wuhan Third Hospital, Tongren Hospital of Wuhan University, Wuhan, China; ^2^Department of Psychiatry, Renmin Hospital of Wuhan University, Wuhan, China; ^3^Medical College of Wuhan University of Science and Technology, Wuhan, China; ^4^Department of Sleep and Psychosomatic Medicine Center, Taihe Hospital, Affiliated Hospital of Hubei University of Medicine, Shiyan, China; ^5^Clinical College of Wuhan University of Science and Technology, Wuhan, China; ^6^Department of Anesthesia, Hubei Provincial Hospital of Traditional Chinese Medicine, Wuhan, China; ^7^Department of Anesthesia, Affiliated Hospital of Hubei University of Traditional Chinese Medicine, Wuhan, China; ^8^Department of Anesthesia, Hubei Province Academy of Traditional Chinese Medicine, Wuhan, China

**Keywords:** degree centrality, migraine, fMRI, support vector machine, biomarker

## Abstract

**Objective:**

Misdiagnosis and missed diagnosis of migraine are common in clinical practice. Currently, the pathophysiological mechanism of migraine is not completely known, and its imaging pathological mechanism has rarely been reported. In this study, functional magnetic resonance imaging (fMRI) technology combined with a support vector machine (SVM) was employed to study the imaging pathological mechanism of migraine to improve the diagnostic accuracy of migraine.

**Methods:**

We randomly recruited 28 migraine patients from Taihe Hospital. In addition, 27 healthy controls were randomly recruited through advertisements. All patients had undergone the Migraine Disability Assessment (MIDAS), Headache Impact Test – 6 (HIT-6), and 15 min magnetic resonance scanning. We ran DPABI (RRID: SCR_010501) on MATLAB (RRID: SCR_001622) to preprocess the data and used REST (RRID: SCR_009641) to calculate the degree centrality (DC) value of the brain region and SVM (RRID: SCR_010243) to classify the data.

**Results:**

Compared with the healthy controls (HCs), the DC value of bilateral inferior temporal gyrus (ITG) in patients with migraine was significantly lower and that of left ITG showed a positive linear correlation with MIDAS scores. The SVM results showed that the DC value of left ITG has the potential to be a diagnostic biomarker for imaging, with the highest diagnostic accuracy, sensitivity, and specificity for patients with migraine of 81.82, 85.71, and 77.78%, respectively.

**Conclusion:**

Our findings demonstrate abnormal DC values in the bilateral ITG among patients with migraine, and the present results provide insights into the neural mechanism of migraines. The abnormal DC values can be used as a potential neuroimaging biomarker for the diagnosis of migraine.

## 1. Introduction

Migraine is a nervous system disease characterized by high attack frequency and disability, which seriously endangers people's physical and mental health and quality of life (1). In recent years, the incidence of the disease has been increasing gradually, thereby affecting patients' normal life and work performance (2). The epidemiological survey of headaches in China in 2010 showed that the incidence of migraine in China was 9.3%. However, the pathophysiological mechanism of migraine is not completely understood, and the accuracy of its early diagnosis is low (3). In addition to symptomatic diagnosis, it is necessary to develop auxiliary diagnostic tools for migraine.

Resting-state functional magnetic resonance imaging (rs-fMRI) is a tool with the advantages of non-invasive and high repeatability, which can reveal the spontaneous activity of brain neurons at the resting state (4, 5). The most commonly used method to detect the spontaneous activity of brain regions is the measurement of blood oxygen level-dependent (BOLD) signals based on differences in magnetic properties of oxyhemoglobin (diamagnetic) and deoxyhemoglobin (paramagnetic). In the brain, blood flow and oxygenated hemoglobin fluctuate with the neuron activity, and thus changes in the BOLD signal can be recorded. Over the years, the BOLD-fMRI technology has been widely used to study neuropsychiatric brain diseases, such as temporal lobe epilepsy (6, 7), depression (8, 9), schizophrenia (10, 11), and mild cognitive impairment (12). Thus, fMRI can be used to study the potential imaging mechanisms of various neuropsychiatric diseases.

With the development of high-definition resolution magnetic resonance technology, it has been found that patients with migraine not only manifest organic changes in brain structure (13) but also show changes in functional connectivity after treatment (14). Recent studies have found that the topological properties of brain networks in patients with episodic migraine are altered (15, 16). Among such properties, the imbalance in the topological structure of brain networks in female migraine patients is the most pronounced (15, 17), but there are few studies that have explored the degree centrality of brain networks involved in migraine. Previous rs-fMRI studies revealed a significant difference in the communication between the brain Island, frontal cortex, and apex nucleus (18–20). Although the results are not consistent, it has been shown that migraines are highly communicative, but other studies found that the communication between migraines is low. To solve this problem, we used the intuitive index of the connection state of each spatial unit of the brain. The number of connections between the voxel in stationary fMRI and other voxels in the brain was calculated, and the pattern classification analysis was performed using the degree centrality (DC). A fully automatic program using 8 min of static fMRI can easily obtain DC and is not limited to the selection of priori region or the network definition. Therefore, it is reliable and can be obtained without the need for artificial estimation and image editing features and is widely used to quantify global connectivity. In recent years, researchers have applied DC to conduct clinical research on neuropsychiatric diseases, such as stroke, depression, schizophrenia, and epilepsy (6, 21–23). Three recent studies reported that migraine patients have abnormal DC values in the whole-brain network. Moreover, a significant between-group difference in DC was found using a data-driven method. Using a data-driven method, Lee et al. found that patients with migraine have altered DC values (24). Other studies found that patients with migraines without aura exhibited significantly smaller DC values in the primary somatosensory cortex and right premotor cortex (25). Moreover, pain intensity is positively correlated with DC in the right amygdala (26). These findings demonstrate differences between migraine patients and bipolar disorder (BD) brain imaging. However, few studies have investigated indicators for the diagnosis, and there are no ideal neuroimaging biomarkers and predictive indicators for the clinical management of migraines. This can be ascribed due to the lack of strict selection of sample size, small sample size, and low sensitivity of analytical methods. Therefore, it is imperative to use DC patterns to explore potential biomarkers for neuroimaging of migraines. The innovation of our study lies in the combination of support vector machine (SVM) and DC analysis methods to identify migraine patients and healthy controls (HCs).

Based on previous studies, we constructed a resting-state brain function network by combining the DC with SVM analysis technology. The differences in DC between migraine patients and HCs were explored to reveal the characteristics of the migraine brain network and find a new method in order to improve the diagnosis of migraine.

## 2. Materials and methods

### 2.1. Subjects

In total, 30 patients with migraine were admitted to the outpatient department and ward of the Department of Psychology of Taihe Hospital in Shiyan from December 2018 to January 2022. The enrolled patients underwent neuropsychological scale assessment and brain resting-state functional magnetic resonance scanning. Among them, 1 patient refused magnetic resonance scanning, and 2 patients were excluded because their head movements were greater than 2 mm during the scanning. Therefore, 27 patients with migraine were enrolled in the study. Inclusion criteria for migraine patients were as follows: (1) The migraine diagnosis was confirmed by the Neurology Department according to the International Headache Society (HIS) criteria (27); (2) right handed; (3) aged 18–60 years; (4) migraine attack interval: no headache attack 3 days before the scan, the day of the scan, and the day after the scan; and (5) sign the relevant informed consent form and voluntarily agree to participate in the research. Exclusion criteria of migraine patients were as follows: (1) previous chronic physical diseases, including cardiovascular and cerebrovascular diseases, hypertension, hyperlipidemia, diabetes, tumors, other types of headache, and other chronic visceral or somatic pain; (2) having a history of chronic mental diseases, such as severe anxiety and depression disorder, sleep disorder, and schizophrenia; (3) history of heroin abuse, alcohol, and other drugs; (4) contraindication of MRI; and (5) pregnancy or lactation. For HCs, 30 healthy volunteers who were matched for the age and gender of migraine patients were enrolled, 2 of whom refused to undergo MRI scanning. Finally, 28 HCs were included in this study. The inclusion criteria of HCs were in line with items 2, 3, and 5 of the inclusion criteria for migraine patients, and the exclusion criteria were in line with items 1, 2, 3, 4, and 5 of the exclusion criteria for migraine patients.

### 2.2. Scale evaluation

Migraine Disability Assessment (MIDAS) and Headache Impact Test – 6 (HIT-6) were used to evaluate the headache and quality of life of all subjects. The MIDAS questionnaire was designed to assess headache-related disabilities and to improve migraine care. The headache patient answered five questions and scored the number of days with limited activity due to migraine in the past 3 months (28). The HIT-6 measures domains related to pain, social functioning, role functioning, vitality, cognitive functioning, and psychological distress (29). The evaluation was performed by two professionally trained graduate students. The study was approved by the ethics committee of the Taihe Hospital, Hubei Medical College, and all participants signed written informed consent forms.

### 2.3. Magnetic resonance imaging scanning procedures

Imaging data were recorded with the Philips 3.0T whole-body MRI scanner. During data acquisition, the participants were required to lie flat in the MRI scanner, relax, close their eyes, and keep their brains awake without any qualitative thinking. Earplugs and eye masks were used to reduce the impact of MRI noise, and foam pads were applied to minimize head movement. All participants were examined by a senior professional MRI technician. Rs-fMRI scanning parameters were as follows: TR = 3,000 ms, TE = 30 ms, matrix = 64 × 64, FOV = 220 × 220 mm, turning angle 90°, slice thickness = 4 mm, slice spacing = 0, a total of 36 layers, scanning the whole brain for 8 min. T1-weighted fast scrambled phase gradient echo sequence was utilized to obtain structural images, with the following scanning parameters: TR = 7.1 ms, TE = 3.5 ms, FOV = 220 × 220 mm, scanning layer thickness = 1 mm, turning angle 8°, matrix 352 × 352, scanning 176 layers. The whole-brain scanning time was 4.2 min.

### 2.4. Data processing

The software package DPABI (RRID: SCR_010501) (30) was run on Matlab 2013a to pre-process the static data as follows: time layer correction, head motion correction, spatial standardization, linear drift, regression to remove covariates (head motion parameters, white matter signals, cerebrospinal fluid signals), and filtering. The images with head translation >2 mm or rotation angle >2° were rescanned on the same day until they met the scientific research needs. The 6 × 6 × 6 mm FWHM Gaussian filter was used to smoothen the image data spatially. Finally, in the REST (RRID: SCR_009641) toolkit, the DC was employed to calculate the *Z*-valued DC distribution map.

### 2.5. Statistical analyses

Statistical analysis was performed using SPSS version 22.0. Gender differences among migraine patients and HCs were compared with the chi-square test; age was tested by two independent samples *t*-test, and a *p*-value of < 0.05 was considered statistically significant. The measurement data were expressed as x ± s. Using gender, age, and years of education as covariates, the REST tool was used to analyze the DC values of migraine patients and HCs by two independent samples *t*-test, and brain regions with altered DC were identified (*P* < 0.01, cluster > 30, GRF correction). The DC values of patients with abnormal brain regions were extracted, and Pearson's correlation analysis was conducted for the neuropsychological scale (*P* < 0.05).

### 2.6. Classification analyses

The SVM was conducted using a library for SVM (LIBSVM, RRID: SCR_010243) software package in Matlab (RRID: SCR_001622). The LIBSVM classifier is trained to learn differences between groups by providing examples in form of (xi, ci), where x represents the DC values of abnormal clusters and c standards for the class label. The grid search method and Gaussian radial basis function kernels were used for parameter optimization. The “leave-pair-out” cross-validation approach was applied using the LIBSVM software to achieve the highest sensitivity and specificity (31).

## 3. Results

### 3.1. Demographic data

A total of 28 migraine patients and 27 HCs were enrolled in the study. There was no significant difference in gender and age between migraine patients and HCs (Table 1).

**Table 1:** Demographic characteristics of the study groups.

**Characteristics**	**Patients (*n* = 28)**	**Controls (*n* = 27)**	**x2 or T**	***P* value**
Gender (male/female)	12/16	12/15	0.89	0.42^a^
Age (years)	38.11 ± 6.49	37.37 ± 5.96	0.48	0.66^b^
MIDAS (scores)	160.17 ± 26.45			
HIT-6 (scores)	61.54 ± 10.11			

a The *p* value for gender distribution was obtained by chi-square test.

b The *p* value were obtained by two sample *t-*tests. MIDAS, Migraine Disability Assessment; HIT-6, Headache Impact Test-6.

### 3.2. DC analysis and correlation analysis

**Figure 1:**
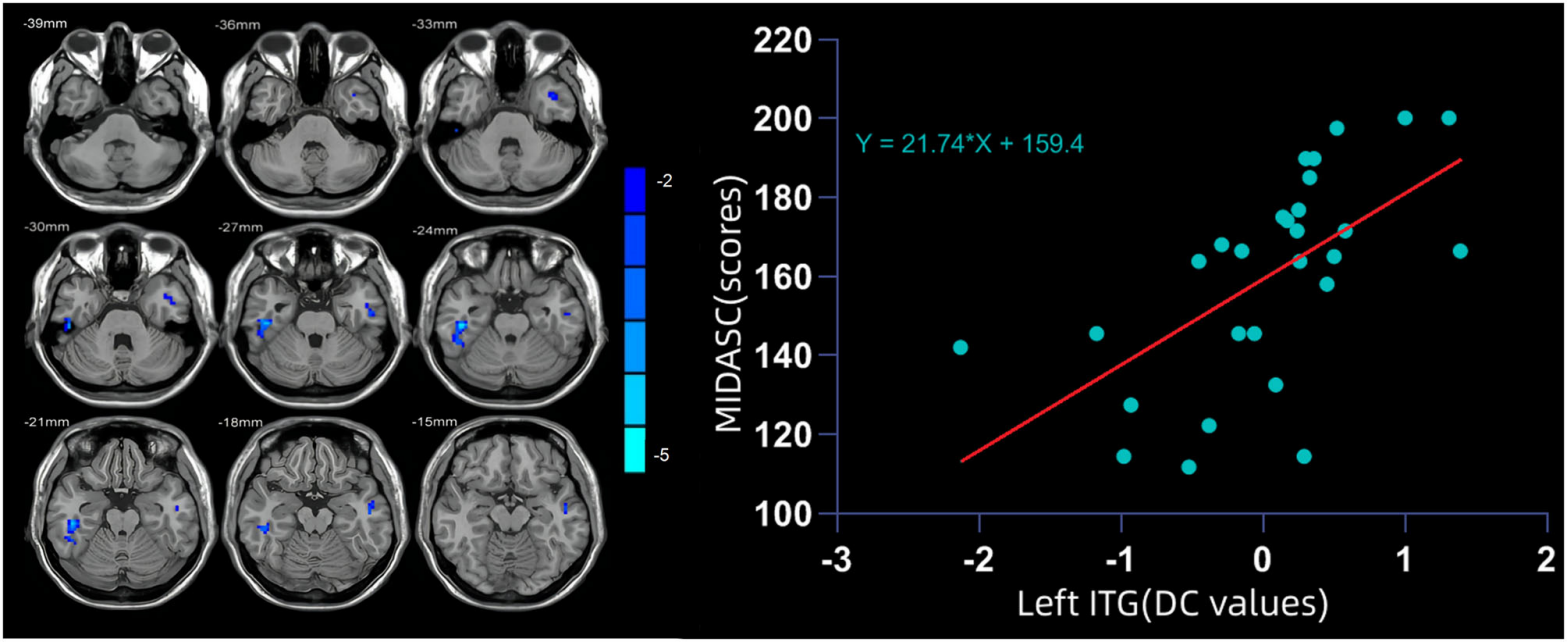
**(Left)** The degree centrality (DC) value of the bilateral inferior temporal gyrus (ITG) in migraine patients was significantly lower than that in healthy controls (HCs). **(Right)** DC value of the left ITG showed a positive linear correlation with MIDAS scores.

Compared with HCs, the DC value of the bilateral ITG in migraine patients was significantly lower and that of left ITG showed a positive linear correlation with MIDAS scores (r = −0.37, *P* < 0.05) (Figure 1 and Table 2).

**Table 2:** Alterations of DC between patients and controls.

**Cluster location**	**Peak (MNI)**			**Number of voxels**	***T*-value**
	***X***	***Y***	***Z***		
Left ITG	−54	−12	−27	37	−3.18
Right ITG	45	−24	−24	87	−4.49

DC, degree centrality; ITG, inferior temporal gyrus.

### 3.3. Support vector machine results

**Figure 2:**
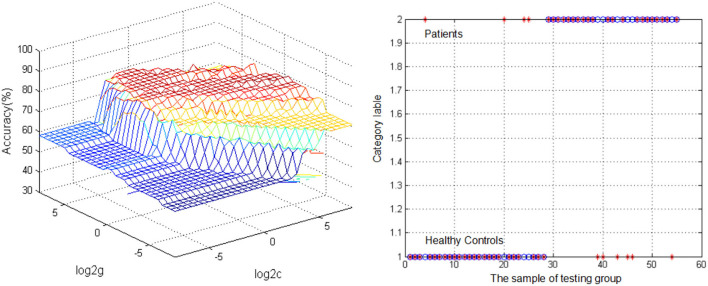
Visualization of classifications through support vector machine (SVM) using the reduced DC values in the left inferior temporal gyrus (ITG) to discriminate migraine patients from healthy controls. **(Left)** SVM parameters result of 3D view. **(Right)** Classified map of the DC values in the left ITG. *Represents the subject.

The SVM analysis was conducted to determine whether DC values in bilateral ITG can be used to discriminate patients from HCs with optimal sensitivity and specificity (Figure 2). The best results were achieved for DC values in the left ITG. This showed a sensitivity of 85.71%, a specificity of 77.78%, and an accuracy of 81.82%.

## 4. Discussion

This study aimed to examine the DC between global network in patients with migraine and HCs. Reduced DC distribution primarily occurred in the bilateral ITG. We also observed that decreased DC in the left ITG was significantly correlated with MIDAS deficits. The SVM classification result suggested that the decreased DC in the left ITG may be an effective indicator for distinguishing patients with migraine from HCs with the highest sensitivity, specificity, and accuracy.

The results of the DC approach indicated that regions with aberrant cooperation were mainly located in the bilateral ITG. Previous studies reported that continuous pain stimulation can change the brain microstructure, and this outcome may be reversible (32). The anatomical distance of the pathway or the changes in connectivity across some brain regions may also gradually destroy the cerebral cortex, thereby affecting the entire brain network (33). A prospective study found that the frequency of migraine attacks in migraine patients increased yearly and that the density of gray matter in some brain regions, such as the hippocampus and supramarginal gyrus, decreased significantly compared with 1 year ago, suggesting that persistent pain can interfere with the brain's processing of information, leading to changes in brain structure (34). These findings demonstrate that patients with migraine manifest an abnormal brain structure and brain function to some extent. Furthermore, transcutaneous vagus nerve stimulation can increase the functional connectivity between the ITG and the anterior cingulate cortex/medial pre-frontal cortex and decrease the connectivity between the thalamus subregion and the precuneus (35). Lai et al. found that the GM of the anterior cingulate cortex of chronic migraine (CM) patients after trauma decreased. During the 12 month follow-up, they found that when the pain disappeared, the gray matter of the thalamus and cerebellum increased, suggesting that the pain processing structure showed adaptive gray matter changes in terms of neuronal plasticity (36, 37). Among these regions, the dorsal anterior cingulate gyrus was significantly related to the course of the disease and was selected as the seed point to perform functional connections with other brain regions. A previous study uncovered the functional connection between the bilateral middle temporal gyrus and the seed regions was enhanced in patients with migraine (38). Although these findings are inconsistent, they can be explained by the following three reasons. First, the subtypes of participants in the studies were inconsistent, and the sample size selection process was different among the studies. Second, the analysis methods varied among the studies. Third, the brain is a complex organ, and thus scanning at different stages of the disease will obtain different outcomes. Nevertheless, findings from these studies provide valuable information that the brain structure and function of migraine patients exhibit some abnormalities.

We also found that the DC values in the left ITG were positively correlated with MIDAS scores. Sequal and colleagues found that the ITG is abnormally activated in patients with migraine in both task-related and resting state (39–41). Therefore, the present findings demonstrated abnormal activity in the ITG and revealed new insights into the pathomechanisms of migraine patients. The reduced neural activity in the left ITG showed the potential to be an indicator for diagnostic neuroimaging of migraine with a high diagnostic accuracy of 81.82%, a sensitivity of 85.71%, and a specificity of 77.78%. This result implies that ITG may be involved in the pathophysiological mechanism of migraine.

## 5. Limitations

There are several limitations to this study. First, a small sample size was enrolled, and most patients were using drugs for symptomatic treatment. Such drugs may affect brain function and hence will affect the outcomes of this study (11, 42). In the future, we will enroll a large sample size and recruit participants who not using drugs to further validate the present findings. Second, the participants were enrolled at one hospital in one region. We plan to recruit participants from multiple centers in the future. Finally, we did not classify migraine patients into subtypes. Different subtypes of migraine may have different imaging mechanisms (43, 44). Therefore, the imaging mechanisms of different subtypes of migraines should be explored in the future.

## 6. Conclusion

This study reported the changes in the bilateral ITG of patients with migraine. The results suggest that normal DC values in the left ITG can be applied in the clinical diagnosis of migraine.

## Data availability statement

The raw data supporting the conclusions of this article will be made available by the authors, without undue reservation.

## Ethics statement

The study was approved by the Ethics Committee of the Taihe Hospital, Hubei Medical College, and all subjects signed the written informed consent. The patients/participants provided their written informed consent to participate in this study. Written informed consent was obtained from the individual(s) for the publication of any potentially identifiable images or data included in this article.

## Author contributions

QW and LX wrote the manuscript. XW, HL, XL, and YZ collected and analyzed the data. YG and CH conceived and critically reviewed the manuscript. All authors have read and approved the final manuscript.

## References

[1] Attack Postdrome PAM. Migraine Episodes Can Be Divided into Several Phases: Prodrome (Also Known as Premonitory), Aura, Migraine Attack and Postdrome. Nat Rev Dis Primer. (2022) 8:1. 10.1038/s41572-022-00335-z35027561

[2] Oie LR Kurth T Gulati S Dodick DW. Migraine and risk of stroke. J Neurol Neurosurg Psychiatry. (2020) 91:593–604. 10.1136/jnnp-2018-318254 32217787 PMC7279194

[3] Loder E. Migraine diagnosis and treatment. Prim Care. (2004) 31:277–92. 10.1016/j.pop.2004.02.003 15172507

[4] Bhattacharyya T Gale D Dewire P Totterman S Gale ME McLaughlin S. The clinical importance of meniscal tears demonstrated by magnetic resonance imaging in osteoarthritis of the knee. J Bone Joint Surg Am. (2003) 85:4–9. 10.2106/00004623-200301000-00002 12533565

[5] Gosseries O Demertzi A Noirhomme Q Tshibanda J Boly M Op de Beeck M. Functional Neuroimaging (FMRI, Pet and Meg): what do we measure? Rev Med Liege. (2008) 63:231–7. 18669186

[6] Gao Y Xiong Z Wang X Ren H Liu R Bai B. Abnormal degree centrality as a potential imaging biomarker for right temporal lobe epilepsy: a resting-state functional magnetic resonance imaging study and support vector machine analysis. Neuroscience. (2022) 487:198–206. 10.1016/j.neuroscience.2022.02.004 35158018

[7] Zhou S Xiong P Ren H Tan W Yan Y Gao Y. Aberrant dorsal attention network homogeneity in patients with right temporal lobe epilepsy. Epilepsy Behav. (2020) 111:107278. 10.1016/j.yebeh.2020.107278 32693375

[8] Gao Y Wang M Yu R Li Y Yang Y Cui X. Abnormal default mode network homogeneity in treatment-naive patients with first-episode depression. Front Psychiatry. (2018) 9:697. 10.3389/fpsyt.2018.00697 30618871 PMC6305293

[9] Guo W Cui X Liu F Chen J Xie G Wu R. Increased anterior default-mode network homogeneity in first-episode, drug-naive major depressive disorder: a replication study. J Affect Disord. (2018) 225:767–72. 10.1016/j.jad.2017.08.089 28938513

[10] Gao Y Tong X Hu J Huang H Guo T Wang G. Decreased resting-state neural signal in the left angular gyrus as a potential neuroimaging biomarker of schizophrenia: an amplitude of low-frequency fluctuation and support vector machine analysis. Front Psychiatry. (2022) 13:949512. 10.3389/fpsyt.2022.949512 36090354 PMC9452648

[11] Cui X Deng Q Lang B Su Q Liu F Zhang Z. Less reduced gray matter volume in the subregions of superior temporal gyrus predicts better treatment efficacy in drug-naive, first-episode schizophrenia. Brain Imaging Behav. (2021) 15:1997–2004. 10.1007/s11682-020-00393-5 33033986

[12] Gao Y Zhao X Huang J Wang S Chen X Li M. Abnormal regional homogeneity in right caudate as a potential neuroimaging biomarker for mild cognitive impairment: a resting-state fmri study and support vector machine analysis. Front Aging Neurosci. (2022) 14:979183. 10.3389/fnagi.2022.979183 36118689 PMC9475111

[13] Petrovi F Stojanov D Aracki-Trenki A Petrovi J Petrovi M. Jankovi S. Brain Magnetic Resonance Spectroscopy in Migraine. Acta Med Medianae. (2021) 60:77–87. 10.5633/amm.2021.0210

[14] Zhang Y Huang Y Li H Yan Z Zhang Y Liu X. Transcutaneous auricular vagus nerve stimulation (tavns) for migraine: an fmri study. Reg Anesth Pain Med. (2021) 46:145–50. 10.1136/rapm-2020-102088 33262253

[15] Coppola G Di Renzo A Tinelli E Di Lorenzo C Scapeccia M Parisi V. Resting state connectivity between default mode network and insula encodes acute migraine headache. Cephalalgia. (2018) 38:846–54. 10.1177/0333102417715230 28605972

[16] Lee MJ Park BY Cho S Park H Kim ST Chung CS. Dynamic functional connectivity of the migraine brain: a resting-state functional magnetic resonance imaging study. Pain. (2019) 160:2776–86. 10.1097/j.pain.0000000000001676 31408050

[17] Liu X Huang L Lei L Xu WU Zuneng LU Xiao Z. Advances in research of functional magnetic resonance imaging in migraine patients. Med Recapitul. (2019) 25:149–53. 10.3969/j.issn.1006-2084.2019.01.029

[18] Hadjikhani N Ward N Boshyan J Napadow V Maeda Y Truini A. The missing link: enhanced functional connectivity between amygdala and visceroceptive cortex in migraine. Cephalalgia. (2013) 33:1264–8. 10.1177/0333102413490344 23720503 PMC3797870

[19] Lei M Zhang JJ Neurology DO Hospital Z University W Neurology DO. Advance of the correlation between insula and migraine. Chin J Clin Neurosci. (2017) 25:470–4. 10.3969/j.issn.1008-0678.2017.04.019

[20] Liu HY Chou KH Lee PL Fuh JL Niddam DM Lai KL. Hippocampus and amygdala volume in relation to migraine frequency and prognosis. Cephalalgia. (2017) 37:1329–36. 10.1177/0333102416678624 27919022

[21] Lin H Xiang X Huang J Xiong S Ren H Gao Y. Abnormal degree centrality values as a potential imaging biomarker for major depressive disorder: a resting-state functional magnetic resonance imaging study and support vector machine analysis. Front Psychiatry. (2022) 13:960294. 10.3389/fpsyt.2022.960294 36147977 PMC9486164

[22] Guo X Wang W Kang L Shu C Bai H Tu N. Abnormal degree centrality in first-episode medication-free adolescent depression at rest: a functional magnetic resonance imaging study and support vector machine analysis. Front Psychiatry. (2022) 13:926292. 10.3389/fpsyt.2022.926292 36245889 PMC9556654

[23] Wang H Chen N Kun-Cheng LI Duan XG Radiology DO Hospital X. Degree centrality in the human functional connectome of basal ganlia stroke patients. Chin J Magn Reson Imag. (2016) 7:727–31. 10.12015/issn.1674-8034.2016.10.002

[24] Lee MJ Park BY Cho S Kim ST Park H Chung CS. Increased connectivity of pain matrix in chronic migraine: a resting-state functional MRI study. J Headache Pain. (2019) 20:29. 10.1186/s10194-019-0986-z 30909865 PMC6734233

[25] Zhang J Su J Wang M Zhao Y Zhang QT Yao Q. The sensorimotor network dysfunction in migraineurs without aura: a resting-state fMRI study. J Neurol. (2017) 264:654–63. 10.1007/s00415-017-8404-4 28154971

[26] Ke J Yu Y Zhang X Su Y Wang X Hu S. Functional alterations in the posterior insula and cerebellum in migraine without aura: a resting-state MRI study. Front Behav Neurosci. (2020) 14:567588. 10.3389/fnbeh.2020.567588 33132860 PMC7573354

[27] Headache Classification Committee of the International Headache S. The International Classification of Headache Disorders, 3rd Edition (Beta Version). Cephalalgia. (2013) 33:629–808. 10.1177/0333102413485658 23771276

[28] Stewart WF Lipton RB Dowson AJ Sawyer J. Development and testing of the migraine disability assessment (midas) questionnaire to assess headache-related disability. Neurology. (2001) 56:S20–8. 10.1212/WNL.56.suppl_1.S20 11294956

[29] Kosinski M Bayliss MS Bjorner JB Ware JE Garber WH Batenhorst A. A six-item short-form survey for measuring headache impact: The Hit-6. Qual Life Res. (2003) 12:963–74. 10.1023/A:1026119331193 14651415

[30] Yan C Zang Y. DPARSF: a MATLAB toolbox for “pipeline” data analysis of resting-state fMRI. Front Syst Neurosci. (2010) 4:13. 10.3389/fnsys.2010.00013 20577591 PMC2889691

[31] Chang CC Lin CJ. LIBSVM: a library for support vector machines. ACM Trans Intell Syst Technol. (2011) 2:1–27. 10.1145/1961189.1961199

[32] Niddam DM Lai KL Tsai SY Lin YR Chen WT Fuh JL. Neurochemical changes in the medial wall of the brain in chronic migraine. Brain. (2018) 141:377–90. 10.1093/brain/awx331 29236991

[33] Zhang X Wang ZH Geng ZJ Zhang Y Zhang L Neurology DO. Functional abnormalities in migraine with aura patients: a resting-state fmri study. J Brain Nerv Dis. (2016) 24:7–11.

[34] Wang Y Zhang Y Luo W. Effect of transcutaneous auricular vagus nerve stimulation on fractional amplitude of low-frequency fluctuation in migraine without aura. J Clin Radiol. (2019) 38:2010–4. 10.13437/j.cnki.jcr.2019.11.002

[35] Redgrave JN Moore L Oyekunle T Ebrahim M Falidas K Snowdon N. Transcutaneous auricular vagus nerve stimulation with concurrent upper limb repetitive task practice for poststroke motor recovery: a pilot study. J Stroke Cerebrovasc Dis. (2018) 27:1998–2005. 10.1016/j.jstrokecerebrovasdis.2018.02.056 29580658

[36] Riederer F Marti M Luechinger R Lanzenberger R von Meyenburg J Gantenbein AR. Grey matter changes associated with medication-overuse headache: correlations with disease related disability and anxiety. World J. Biol. Psychiatry. (2012) 12:517–25. 10.3109/15622975.2012.665175. 22746999

[37] Lai TH Chou KH Fuh JL Lee PL Kung YC Lin CP. Gray matter changes related to medication overuse in patients with chronic migraine. Cephalalgia. (2016) 36:1324–33. 10.1177/0333102416630593 26853805

[38] Gomez-Beldarrain M Oroz I Zapirain BG Ruanova BF Fernandez YG Cabrera A. Right fronto-insular white matter tracts link cognitive reserve and pain in migraine patients. J Headache Pain. (2015) 17:4. 10.1186/s10194-016-0593-1 26830863 PMC4735096

[39] Chen C Yan M Yu Y Ke J Xu C Guo X. Alterations in regional homogeneity assessed by fMRI in patients with migraine without aura. J Med Syst. (2019) 43:298. 10.1007/s10916-019-1425-z 31352647

[40] Schwedt TJ Chiang CC Chong CD Dodick DW. Functional MRI of migraine. Lancet Neurol. (2015) 14:81–91. 10.1016/S1474-4422(14)70193-0 25496899 PMC11318354

[41] Crowell GF Stump DA Biller J McHenry LC Toole JF. The transient global amnesia-migraine connection. Arch Neurol. (1984) 41:75–9. 10.1001/archneur.1984.04050130081029 6689894

[42] Guo W Liu F Chen J Wu R Li L Zhang Z. Olanzapine modulates the default-mode network homogeneity in recurrent drug-free schizophrenia at rest. Aust N Z J Psychiatry. (2017) 51:1000–9. 10.1177/0004867417714952 28605934

[43] Pisanu C Lundin E Preisig M Gholam-Rezaee M Castelao E Pistis G. Major depression subtypes are differentially associated with migraine subtype, prevalence and severity. Cephalalgia. (2020) 40:347–56. 10.1177/0333102419884935 31645113

[44] Si N Xu H Mao JH Li L Gu W. Relationships of different migraine subtypes and severity of balance disorder. China J Mod Med. (2018) 28:104-8. 10.3969/j.issn.1005-8982.2018.23.023

